# Characterization of tinnitus in the elderly and its possible related disorders

**DOI:** 10.1016/S1808-8694(15)30786-2

**Published:** 2015-10-19

**Authors:** Lidiane Maria de Brito Macedo Ferreira, Alberto Novaes Ramos, Eveline Pereira Mendes

**Affiliations:** 1 Otorhinolaryngologist; 2 Doctor and Infectologist - Department of Health. Professor of the Department of Community Health, Faculdade de Medicina da Universidade Federal do Ceará; 3 Speech therapist for hearing aids at the HGF

**Keywords:** elderly, quality of life, tinnitus

## Abstract

Population aging it is a current reality in Brazil and tinnitus appears as a very prevalent symptom, having a high impact on the quality of life of elderly patients.

**Aim:**

to evaluate and to characterize tinnitus in this group.

**Materials and Methods:**

A research questionnaire randomly given to 100 elderly patients in a tertiary hospital, asking about tinnitus characteristics, its impact on the life of the patient, and personal medical history.

**Results:**

61% of the participants were female, average age average was 69.53 years.

**Conclusions:**

Tinnitus has a relevant impact on the lives of the elderly; there was no correlation between the level of hearing loss and the level of patient dissatisfaction caused by tinnitus; and presbycusis was the most common finding in the audiometric tests.

## INTRODUCTION

Population aging is a worldwide reality, even in underdeveloped scenarios. According to the Brazilian law number 8 842 of 04 January 1994, an elderly person is defined as an individual aged over 60 years. The World Health Organization defines the elderly in developing countries as persons aged 60 years or over, and in developed countries as persons aged 65 years or over.^1^ The worldwide life expectancy at birth was estimated in 2000 as being 65 years; for 2045–2050, the United Nations estimates a life expectancy of 74.3 years.^2^

Among the morbidities of the elderly, tinnitus is clinically relevant, since, although being a symptom rather than a disease, it can affect individual functioning as a whole. Tinnitus affects 15% of the general population and 33% of elderly persons.^3^ It is an extremely frequent disorder that affects about 40 million persons in the US, of whom 10 million are affected severely.^4^ Tinnitus may be caused by numerous otological, metabolic, neurological, orthopedic, cardiovascular, pharmacological, dental, and psychological conditions, more than one of which may be present in the same individual.^5^^,^^6^

Sataloff et al.^4^ reported that about 85% of patients visiting an otologist have tinnitus. Benevides^7^ reported that tinnitus often accompanies presbyacusis, and may be even more troublesome than deafness. There are no absolute numbers on the prevalence of tinnitus in Brazil; however, it has been estimated that these numbers are similar to those in developed countries. The reason for this is that the epidemiology of elderly patients in Brazil is similar to that of elderly persons in First world countries in term of the presence of chronic-degenerative diseases (which, as mentioned above, are closely related with the origin of tinnitus).

In 2004, a study in Fortaleza of 260 elderly patients with auditory complaints revealed that tinnitus was the most prevalent complaint (58.08%), which confirms its frequency in elderly patients seeking otorhinolaryngological help.^8^

The presence of tinnitus often causes many negative repercussions on an individual's life; sleep, concentration on daily and professional activities, social life, and emotional balance may all be affected. Anxiety and depression may ensue.^5^^,^^6^^,^^9^, ^10^, ^11^

The psychological aspects of tinnitus are well known and have been discussed by many authors.^12^, ^13^, ^14^, ^15^ According to Sanchez,^16^ the presence of hearing loss increases the risk of tinnitus affecting concentration and emotional balance as a cofactor.

This study originated from the clinical observation that many elderly patients manifest tinnitus as a cause of distress and severe health issues in general, resulting in inability to carry out daily activities. The reason for this study, therefore, were: a high prevalence of tinnitus in the elderly;^3^ the fact that tinnitus is considered as a cause of poor intelligibility in this population group, affecting interpersonal relationships; the presence of behavioral symptoms such as irritability and insomnia, which may lead to depression; and the fact that tinnitus may affect activities of daily living (ADL) and instrumental activities of daily living (IADL).

The purposes of this study were: to study the epidemiology of elderly persons with tinnitus seen at a tertiary hospital in Fortaleza, CE; to characterize tinnitus in these elderly patients; to correlate existing comorbidities in elderly patients with tinnitus; to identify audiometric findings in elderly patients with tinnitus; and to quantify the effect of tinnitus on the lives of elderly persons.

**MATERIAL AND METHOD**

A cross-sectional, descriptive, quantitative study was undertaken based on the otorhinolaryngology outpatient unit of a tertiary hospital in Fortaleza, Ceara, which is a reference for this medical specialty. Participants were elderly patients (age over 60 years) with tinnitus, who sought medical care at the hospital, regardless of the medical specialty. These elderly patients were invited by posters placed along the hospital aisles about the study. The first 100 elderly patients of both sexes that visited the otorhinolaryngology outpatient unit and who consented to participate were included. Exclusion criteria were: patients with tinnitus due to acute conditions, such as acute otitis or earwax, and persons with difficulty in performing audiometry (cognitive deficits or inconsistent responses). The study period was from June to December 2006. As these patients presented to the otorhinolaryngology outpatient unit, a second history was taken and a basic otorhinolaryngological physical examination was done; all were done by the main researcher. Patients were scheduled for audiometry, which was carried out by a single speech therapist for standardization. A standard questionnaire adapted from Sanchez^16^ was applied; it consisted of questions on: age and sex, features of tinnitus (type, frequency of perception, location, and onset), the effect of tinnitus on the patient's life, and a clinical history.

After these tests, the elderly patients were again seen by the main researcher; depending on the findings, patients were referred for specific therapy within the hospital.

Audiometry was done by the same speech therapist using an Interacoustics model AD-28, audiometer in an acoustic cabin. Davis and Silverman's^17^ classification was applied for evaluating hearing loss.

In cases where the audiometric curves were asymmetric, other tests were carried out, such as the brainstem evoked potential and cranial magnetic resonance imaging, to exclude retrocochlear causes.

Data analysis was done using descriptive statistics and context analysis to describe the variables and build tables and charts. The software Microsoft Office Excel® was used for making the data spreadsheets, tables and charts. Subjects were enrolled in this study after understanding all steps involved in participation and after signing a free informed consent form. The Research Ethics Committee of the hospital evaluated and approved the study on 01 June 2006 (number 010604/06).

## RESULTS

**Chart 1:**
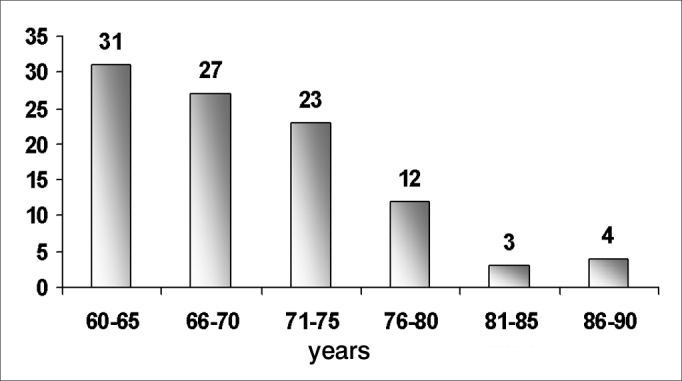
Percentage distribution of tinnitus patients (n=100) according to age

**Chart 2:**
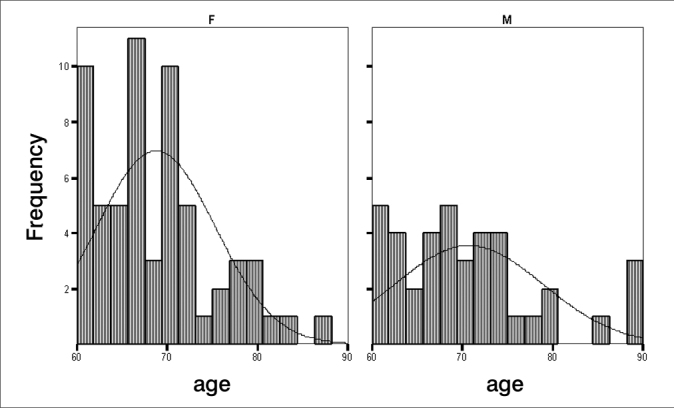
Age distribution of tinnitus patients (n=100) according to sex

**Chart 3:**
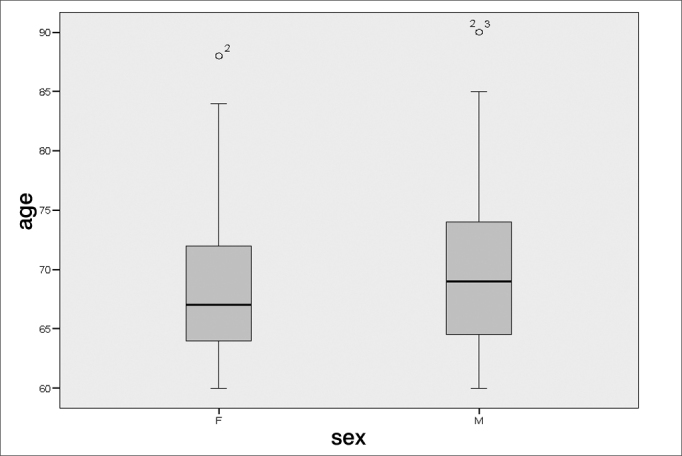
Age distribution analysis of tinnitus patients (n=100) according to sex

There were more elderly females with tinnitus (61.0%) within the 60 to 70 year range among the 100 patients of the sample (Frame 1, Chart 1, Chart 2, Chart 3).

**Frame 1:** Descriptive statistics. Age according to sex.

	sex		Stat.	Standard error
Age	F	Mean	68,77	,839
			Lower Limit	67,09
		Confidence Interval (95%) for the mean		
			Upper Limit	70,45
		Median	67,00	
		Variance	42,913	
		Standard Deviation	6,551	
		Minimum	60	
		Maximum	88	
		Variation	28	
		Interquartile Var.	9	
		Skewness	,711	,306
		Kurtosis	,141	,604
	M	Mean	70,72	1,315
		Confidence Interval (95%) for the mean	Lower Limit	68,06
			Upper Limit	73,38
		Median	69,00	
		Variance	67,418	
		Standard Deviation	8,211	
		Minimum	60	
		Maximum	90	
		Variation	30	
		Interquartile Var.	10	
		Skewness	,912	,378
		Kurtosis	,470	,741

Tinnitus was characterized by patients mostly as being non-pulsatile, continuous, bilateral, recent, and discrete (Table 1).

**Table 1:** Features of tinnitus in the sample of elderly subjects (n=100).

Features of Tinnitus	%
Type	
- Pulsatile	24,0
- Non-pulsatile	76,0
Frequency of Perception	
-Intermittent	46,0
-Continuous	54,0
Location	
-Unilateral	43,0
-Bilateral	57,0
Time	
-Recent	62,0
-Mid-term	17,0
-Long term	21,0
Type	
-Discrete	83,0
-Multiple	17,0

**Chart 4:**
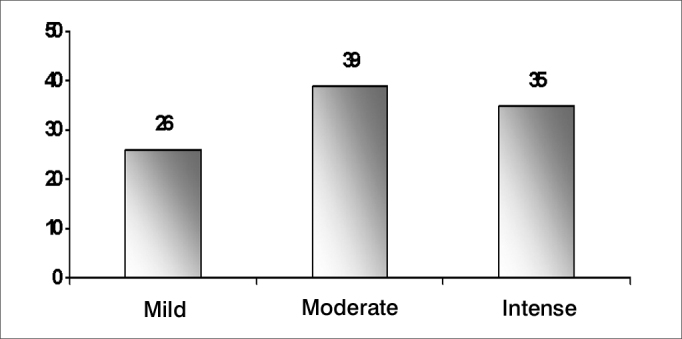
Percentage distribution of how disturbed elderly patients were with tinnitus according to the visual-analog scale (n=100)

The effect of tinnitus was measured based on information about interferences on daily situations and moments, as shown on Table 2 and Chart 4.

**Table 2:** Distribution frequency of tinnitus as to its repercussion on the lives of elderly patients (n=100).

REPERCUSSION	N	% (of total interference)
Interferes with sleep	41	31.8
Interferes with concentration	29	22.5
Interferes emotionally	42	32.5
Interferes with social life	17	13.2

As mentioned above, many factors may cause tinnitus; in this study, the main such factors were arterial hypertension and use of medication (Table 3).

**Table 3:** Risk factors associated with the presence of tinnitus in elderly patients (n=100).

RISK FACTOR	%
Hypoacusis	74,00
Use of medication	74,00
Vertigo	52,00
Arterial systolic hypertension	49,00
Diet with stimulants	36,00
Auditory hypersensitivity	30,00
Dyslipidemia	27,00
Diabetes mellitus	12,00
PVD	12,00
Smoking	12,00
Use of alcohol	5,00

**Chart 5:**
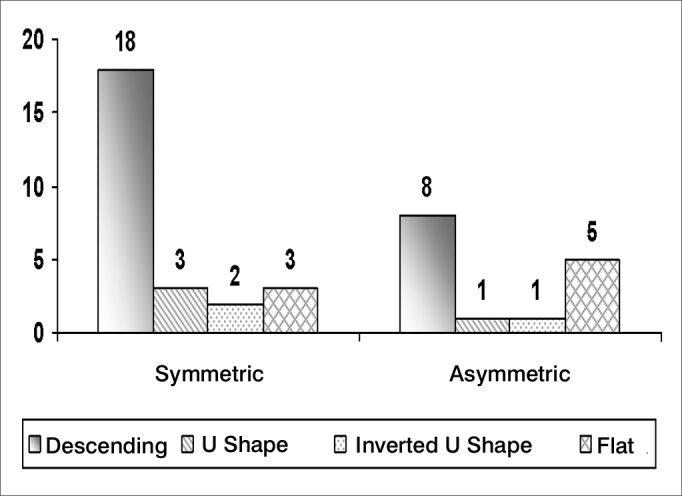
Percentage distribution of an assessment of tinnitus in elderly patients in the visual-analog scale (n=100)

**Chart 6:**
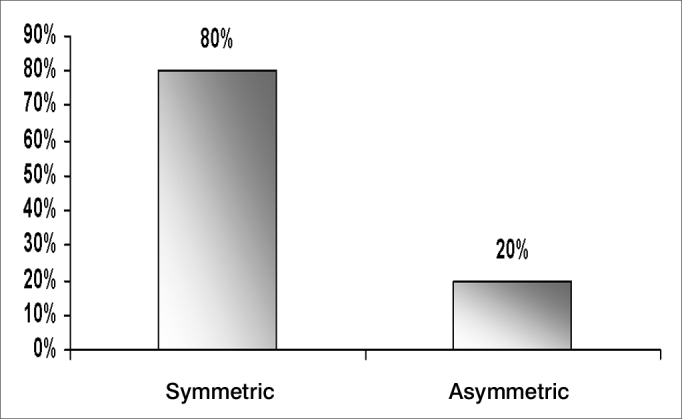
Distribution of audiometric curves according to interaural symmetry in elderly patients

**Chart 7:**
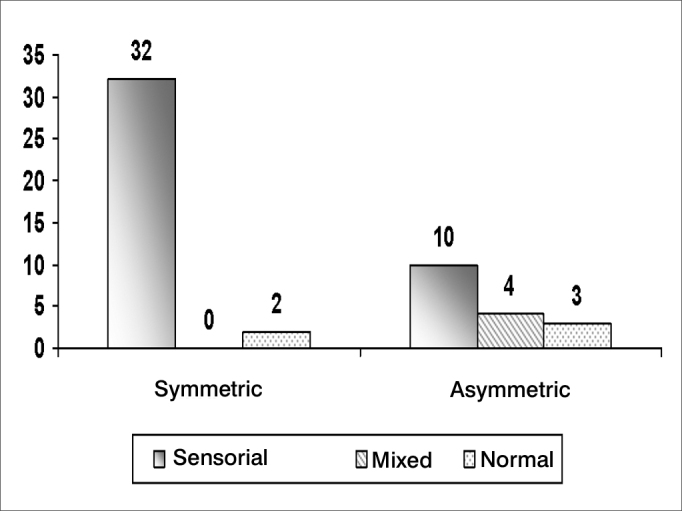
Distribution of audiometric curves according to the nature of hearing loss in elderly patients

Hearing loss is a symptom frequently associated with tinnitus; its features are shown on Chart 5, Chart 6, Chart 7, and Table 4.

**Table 4:** Percentage ratio of interference caused by tinnitus and the degree of hearing loss in elderly patients (n=100).

Tinnitus	Mild loss	Moderate loss and moderately severe loss	Severe and deep loss	No loss	Total
Mild	33.4	40.0	20.0	6.7	100.00
Moderate	23.1	50.0	23.1	3.8	100.00
Intense	48.0	40.0	8.0	4.0	100.00

## DISCUSSION

The series included patients seeking health care in any of the hospital's units; this probably was reflected in the fact that women often seek medical help more frequently than men.

The age ranged from 60 to 90 years (variance – 30); the mean age was 69.53 years, and the standard deviation was 7.26 years, similar to the mean found in other studies (65.5 years).^18^ The median age was 69 years; the mode was 71 years. A study done also in Fortaleza in 20048 showed that the most frequent age group was that from 65 and 70 years, which is similar to our results (Chart 1, Chart 2, Chart 3).

The main features of tinnitus were: non-pulsatile, continuous, bilateral, recent, and discrete (Table 1). Person19 found a 36.0% rate for intermittent tinnitus and a 63.3% rate for continuous tinnitus in his series, confirming our data that continuous tinnitus is more frequent.

When asked about the effect of tinnitus on their daily lives, most of the patients reported difficulty in sleeping and emotional distress (Table 2). This reflects in the score patients attributed to tinnitus - marked, in most cases (Chart 4).

How disturbed patients felt because of tinnitus is a subjective feeling that often depends on external and psychological factors, as well as the negative connotation they attribute to it. We assessed this level of disturbance in a zero to ten scale or in a visual analog scale with sad to happy faces to score the degree of dissatisfaction with tinnitus. There were 39.0% patients complaining of moderate tinnitus, which is fairly similar to other published results (49.0%^18^ and 31.8%^19^). Severe tinnitus was found in 35%, and mild tinnitus was found in 26.0%.

Comorbidities may cause or precipitate tinnitus; a high percentage of elderly patents in our sample made use of medication (some of them using more than one drug) and had arterial hypertension, as well as otological symptoms (Table 3). Clinical practice shows - notwithstanding the lack of concrete data - that changes in blood pressure, blood glucose and zinc levels,^19^ and even dietary compounds (such as xanthines) may lead to tinnitus.

The involvement of arterial hypertension in the genesis of tinnitus remains controversial. Brohen^20^ (1996) analyzed a group of hypertensive patients and found tinnitus in 36.0%; Baraldi21 (2004) correlated tinnitus with hearing loss and found that 34.2% of these patients high blood pressure. In our sample, 49% of the elderly patients were hypertensive. Our assessment revealed a high correlation between tinnitus and arterial hypertension; on the other hand, there was no significant association with diabetes, dyslipidemias, peripheral vascular disease, and stimulants in the diet. Medication was being used for these conditions in 74.0% of patients; the most frequently used drugs were antihypertensives. Antivertigo medication and other centrally-acting drugs, such as antidepressants and benzodiazepines, were also mentioned.

Smoking and use of alcohol did not correlate strongly with tinnitus; these habits were present in 12.0% and 5.0% of cases.

Otoneurological symptoms, which have been studied in detail, are clearly associated with tinnitus. Hypoacusis, vertigo and tinnitus form the classic otoneurological triad. Person^19^ correlated otoneurological symptoms with low blood zinc levels and found a 74% association between hypoacusis and tinnitus and a 52% association between vertigo and tinnitus. Our findings show a 59.1% and a 41.0% association for these symptoms, which are close to the rate of tinnitus in the sample.

Audiometry is an important audiological and otoneurological screening test; most of our sample had symmetrical, sensorineural, descending curves, which is typical of elderly patients with presbyacusis (Chart 5, Chart 6, Chart 7). Symmetry was found in 80.0% of cases, similar to the findings of Amaral^8^ - also from Fortaleza - who found a 73.8% rate.

How disturbed patients are with tinnitus depends also on age-associated hearing loss (presbyacusis); this may lead to social withdrawal, often due to communication difficulties. Attention and concentration deficits and sleep disorders, which are common at this age, gain extra weight in the presence of tinnitus; in this situation, instrumental activities of daily living are harder to perform, and the risk of falls is increased.

Hypoacusis (as a complaint of patients, but not demonstrated audiologically) was highly associated with our cases (74.0%). Vertigo was present in 52.0% of cases, and auditory hypersensitivity was present in 30.0% of cases. The specific type of hypoacusis is directly related with symptoms, since presbyacusis affects labyrinthic and cochlear homeostasis in most cases, and may also cause concomitant generally bilateral vertigo and tinnitus. The possibility of retrocochlear or neural conditions becomes more evident in cases where there is auditory asymmetry or unilateral tinnitus; in these cases, other associated systemic findings should be sought. Our sample confirms that characteristic bilateral sensorineural descending hearing loss - common in presbyacusis - is the main factor associated with tinnitus in the elderly. This is due to the loss of cochlear hair cells with aging. All cases with asymmetrical loss were investigated with cerebral image exams; no retrocochlear diseases were found.

Asymmetrical unilateral or bilateral hearing loss was found in 26.9% of cases; symmetrical bilateral hearing loss was found in 65.4% of cases; and there was no hearing loss in 7.7% of cases. We found no correlation between the degree of hearing loss as measured in audiometry and how disturbing was the tinnitus, showing that the reaction to tinnitus is related with the manner by which patients face this symptom, rather than any physical or anatomical measure (Table 4).

## CONCLUSION

We found that tinnitus causes much dissatisfaction in elderly patients, since this symptom affects their daily activities and may alter sleeping patterns and the emotional status. Tinnitus needs to be approached as a relevant symptom in any treatment. An association with use of many medications should be sought, as well as depression, which may be common in the elderly.

We were also able to correlate the presence of arterial hypertension and tinnitus in these patients, as well as the presence of other otoneurological symptoms. As expected, presbyacusis was the main associated condition on audiometry in this population group. There was no correlation between the audiometric degree of hearing loss and how disturbed patients were with tinnitus. The limbic system, thus, is much more involved in the genesis and maintenance of tinnitus than the auditory system itself.
